# Lentiviral Vector Induced Modeling of High-Grade Spinal Cord Glioma in Minipigs

**DOI:** 10.1038/s41598-020-62167-9

**Published:** 2020-03-24

**Authors:** Muhibullah S. Tora, Pavlos Texakalidis, Stewart Neill, Jeremy Wetzel, Rima S. Rindler, Nathan Hardcastle, Purva P. Nagarajan, Andrey Krasnopeyev, Cristin Roach, Raphael James, Jeffrey N. Bruce, Peter Canoll, Thais Federici, John N. Oshinski, Nicholas M. Boulis

**Affiliations:** 1 Department of Neurosurgery, Emory University School of Medicine, Atlanta, GA USA; 2 Department of Biomedical Engineering, Georgia Institute of Technology, Atlanta, GA USA; 3 Department of Pathology, Emory University School of Medicine, Atlanta, GA USA; 4 Division of Animal Resources, Emory University School of Medicine, Atlanta, GA USA; 5 Department of Neurology, Emory University School of Medicine, Atlanta, GA USA; 6 Department of Neurosurgery, Columbia University, New York, NY USA; 7 Department of Pathology and Cell Biology, Columbia University, New York, NY USA; 8 Department of Radiology, Emory University School of Medicine, Atlanta, GA USA

**Keywords:** CNS cancer, Cancer models

## Abstract

Background: Prior studies have applied driver mutations targeting the RTK/RAS/PI3K and p53 pathways to induce the formation of high-grade gliomas in rodent models. In the present study, we report the production of a high-grade spinal cord glioma model in pigs using lentiviral gene transfer. Methods: Six Gottingen Minipigs received thoracolumbar (T14-L1) lateral white matter injections of a combination of lentiviral vectors, expressing platelet-derived growth factor beta (PDGF-B), constitutive HRAS, and shRNA-p53 respectively. All animals received injection of control vectors into the contralateral cord. Animals underwent baseline and endpoint magnetic resonance imaging (MRI) and were evaluated daily for clinical deficits. Hematoxylin and eosin (H&E) and immunohistochemical analysis was conducted. Data are presented using descriptive statistics including relative frequencies, mean, standard deviation, and range. Results: 100% of animals (n = 6/6) developed clinical motor deficits ipsilateral to the oncogenic lentiviral injections by a three-week endpoint. MRI scans at endpoint demonstrated contrast enhancing mass lesions at the site of oncogenic lentiviral injection and not at the site of control injections. Immunohistochemistry demonstrated positive staining for GFAP, Olig2, and a high Ki-67 proliferative index. Histopathologic features demonstrate consistent and reproducible growth of a high-grade glioma in all animals. Conclusions: Lentiviral gene transfer represents a feasible pathway to glioma modeling in higher order species. The present model is the first lentiviral vector induced pig model of high-grade spinal cord glioma and may potentially be used in preclinical therapeutic development programs.

## Introduction

High-grade glioma has a clinical picture of untenable morbidity and mortality^1,2^. Given this clinical need, the U.S. National Library of Medicine has over 350 completed phase II-III clinical trials registered as of this writing. Unfortunately, there have been limited changes to the clinical outcomes in patients with high-grade glioma over the past years. In part, this represents the malignant nature of a disease that is refractory to a variety of treatment approaches. On the other hand, this raises the question of the translational value of existing pre-clinical animal models as a pathway to the clinic^3,4^. Given numerous strategies with measured preclinical optimism including immunotherapy, oncolytic vectors, and targeted drug delivery, more appropriate large animal model systems are necessary to bridge the translational gap^5–10^.

In patients, high-grade gliomas present with marked invasion along white matter tracts and surrounding parenchyma, an immunosuppressive microenvironment, and significant inter and intra-tumoral heterogeneity^11^. Existing models of high-grade gliomas are variable in recapitulation of these features. The most common are xenograft and syngeneic models which have drawbacks including non-invasive growth (9L, U87, U251), immunogenicity (U251, U87, C6, 9L), and restriction to murine (CT-2A, GL261) and rat models (C6, CNS-1)^12^. One of the most extensively used models for immunotherapeutic studies is the GL261 line. Originally developed in 1939 through administration of methylcholanthrene pellets into the murine brain, it has served as a critical resource given its diffuse infiltration, histopathologic characteristics, and capacity to be employed in wildtype C57BL/6 mice. Unfortunately, since this is a syngeneic mouse model, transplantation of these cannot be scaled into large animals for use a more surgically translatable anatomic space^13^.

Vector-driven glioma models represent an immunocompetent option that could feasibly be scaled up to a large animal model, well reviewed elsewhere^3^. In particular, platelet-derived growth factor Beta (PDGF-B) driven glioma models have been demonstrated *in-silico*, *in-vitro*, and in *in-vivo –* using rodent models with retroviral expression of PDGF-B, HRAS-G12V, shRNA-P53, and other transgenes implicated in the disease pathogenesis^14–19^. In 2011, Lei *et al*. first reported that PDGF-B-driven models recapitulate the proneural glioblastoma subtype^20^. This was further investigated by Sonabend *et al*. in 2014 with robust characterization of the master regulator transcriptional network that drives glial progenitor transformation^21^. As recently as 2018, transcriptomic characterization of mouse models using lentiviral PDGF-B and shRNA-CDK2NA have also been shown to produce pro-neural subtype glioblastoma^22^. In mouse models, it is reported that additional genetic lesions may be required to model high-grade gliomagenesis with either constitutive RAS, or more importantly, knockdown of CDK2NA or p53^22,23^.

In optimizing a model system for high-grade glioma, the use of a large animal system would provide improved utility for translation of neurosurgical strategies, device development, drug delivery, and radiologic study^24–26^. From an anatomic standpoint, the rodent brain is lissencephalic creating a minimization of drug leakage and improvement of local drug delivery^27,28^. Furthermore, the murine brain and spinal cord have significant developmental differences in comparison to large animal systems^27–29^. In addition to these anatomic considerations, the aforementioned concerns with immunocompromised models using xenograft cell lines have generated a translational space that may explain some of the limited success with preclinical strategies^4,25,30^. Moreover, the use of viral vector-driven approaches in, targeting distinct genetic lesions implicated in the human disease, have been widely reported as reliable, highly penetrant, and with striking histopathologic and molecular validity^14,16,17,22^.

The genetic profile, immune system, and the size and anatomy of the porcine brain and spinal cord is recognized to better model the human^25,29,31^. For these reasons, device implantation, stereotactic radiosurgery, and intrathecal and intraparenchymal drug delivery have all been performed in pigs^7,27,32–36^. Furthermore, while the Food and Drug Administration (FDA) has recognized proof-of-principle data on therapeutic efficacy in highly characterized rodent models, the use of large animals is considered critical for clinical relevance. Recognizing these considerations, groups have utilized the U87 xenografts in pigs, but required Cyclosporine-based immunosuppression and continued to face the known limitations of the U87 cell line^27,37^. Overall, existing animal models have limited translational utility due to a confluence of factors including immunogenicity, immunocompromised systems, variable recapitulation of histopathologic features, and limited anatomic translatability, highlighting the significance of generation and validation of an immunocompetent large-animal model.

Thus, the goal of the present study is to scale previous strategies from murine models into a large animal model. Here we describe the use of lentiviral gene transfer to generate an immunocompetent large animal model of high-grade glioma in the porcine spinal cord with subsequent histopathologic, radiologic, and behavioral analysis.

## Materials and Methods

### Vector design

It is well known that HGGs involve a mixed mutational profile with marked heterogeneity between patients. However, the RTK/RAS/PI3K and p14(ARF)/CDKN2A and TP53 pathways are involved in up to 88% and 50% of high-grade gliomas respectively^17^. Therefore, we decided to target these pathways, as has been performed in rodent models, to establish the feasibility of scaling this strategy into a pig model. Three individual lentiviral vectors were designed to target the RTK/RAS/PI3K and TP53 pathways. These vectors were designed separately to avoid the risk of decreased transduction efficiency from larger insert sizes, biosafety requirements, and to provide flexibility to adjust included vectors for future study^38^. Raw sequences of transfer plasmids are presented in Supplemental File 1 and schematics of transgenes in Supplemental Fig. 1.

All lentiviral vectors were VSV-G pseudotyped, third-generation, replication deficient systems titered at >10^9^ infectious units (IU)/ml. Vector 1: PDGF-B-IRES-eGFP and Vector 2: HRASG12V-IRES-mPlum were designed using a pCDH transfer plasmid backbone with an Ef1α promoter and reporters following an internal ribosomal entry site (IRES) sequence. Vector 3 was designed using a pLKO1 backbone and expressed two sequences of shRNA targeting porcine p53 including sequences 787 and 944 under the H1 and U6 promoters, respectively as reported by Merkl *et al*.^39^. These shRNA sequences have 100% homology to two regions of porcine TP53 mRNA and have been used for efficient knockdown in porcine cell culture models^39^. Controls for vectors 1–3 only included reporters and shRNA scramble, referred to as **CTRL**. The oncogenic cocktail will be referred to as **ONC**. All vectors (ONC or CTRL) were thawed and combined in equal parts immediately prior to inoculation.

### Surgical approach

**Figure 1:**
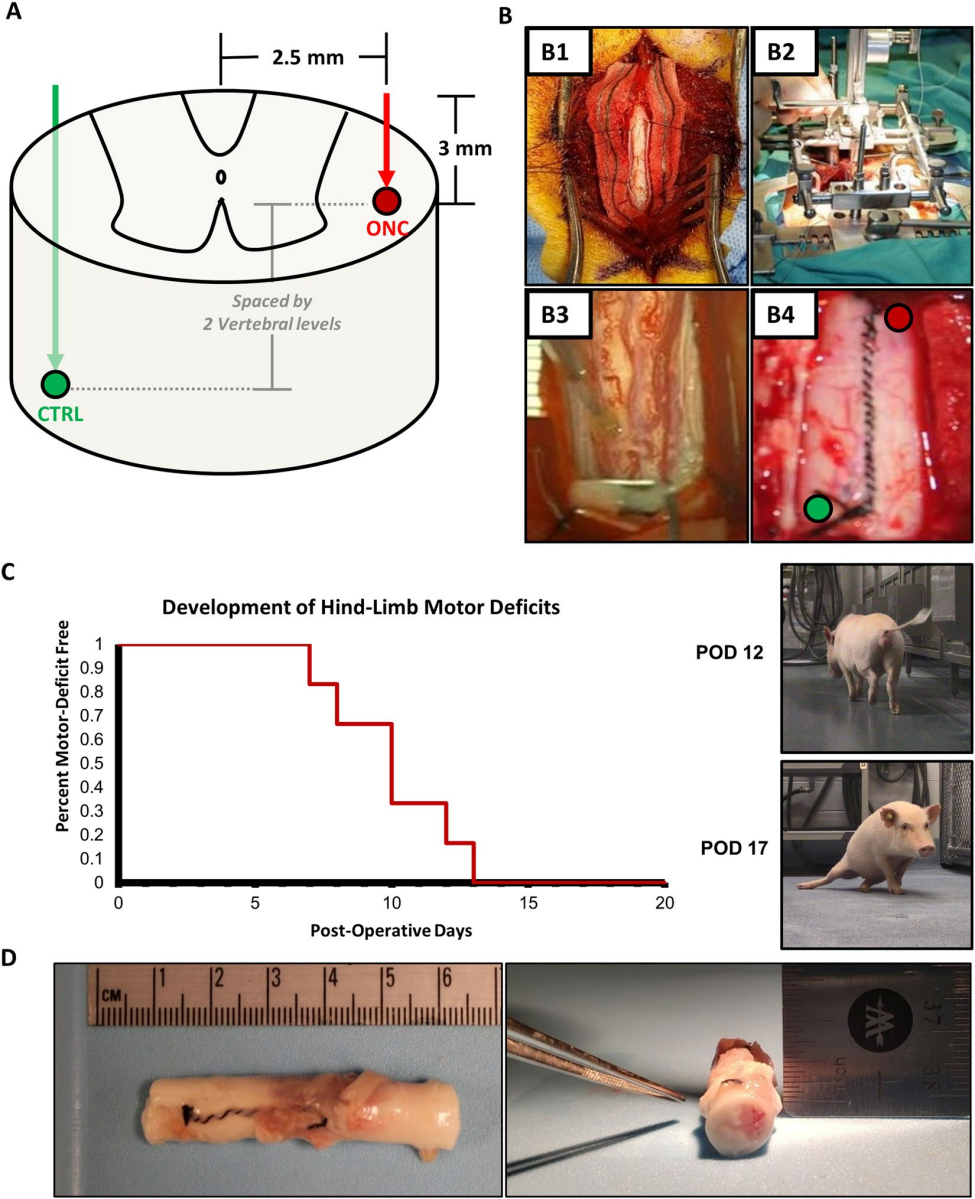
Surgery, Motor Deficits, and Gross Pathology. (**A**) Targeted injection of lateral white matter of the thoraco-lumbar spinal cord. **(B)** Surgical approach with exposure of the spinal cord (B1), mounting of the spinal derrick stereotactic platform (B2), injection (B3), and dural closure (B4). **(C)** All animals developed clinically appreciable hindlimb motor deficits by post-operative day 13 (n = 6/6). **(D)** Gross pathology of spinal cord demonstrates spinal cord expansion and a mass forming lesion at the site of ONC injection and not CTRL (Pig 3 necropsy shown).

The development of an intraparenchymal mass forming lesion, as with a glioma in the spinal cord, would progress in human patients to yield an anatomically localizable neurologic deficit. As such, we selected the thoracolumbar spinal cord of the Gӧttingen minipigs to inject the oncogenic lentiviral vectors. This location of injection provides a distinct advantage, where tumor progression will incur clinically appreciable and quantifiable motor deficits affecting the hind-limb ipsilateral to the site of injection. The surgical procedure was performed as in prior studies and as depicted in Fig. 1A,B^40^. Briefly, the animals underwent a two-level laminectomy and dural exposure. The spinal derrick stereotactic platform was mounted onto the dorsum of the animal for targeting the lateral white matter (2.5 mm lateral to midline, at rostral or caudal positions for each injection, spaced by 2 vertebral levels)^40^. This spacing is necessary to allow for exclusive analysis of the injection sites, space for tumor growth, and delineation of laterality of motor deficits. This intraparenchymal injection strategy is been reported to be safe in pigs and has been implemented in human clinical trials ^40,41^.

All injections were performed at a rate of 2.5 ul/minute, maximal volume of 25 ul, and a 3 mm depth with a 30-gauge stainless-steel needle and MINJ-PD microINJECTOR pump (Tritech Research, Inc., Los Angeles, CA). CTRL and ONC injections were randomized to either rostral or caudal site of injections on contralateral sides, as depicted in Fig. 1A. A 1-minute dwell time was applied before removal of the needle to minimize reflux of the injection. The investigators performing post-operative evaluations were blinded to CTRL and ONC assignments.

### Animals, physical examination, and behavior

Our laboratory has previously used the Gӧttingen minipig as optimal systems for scaling vector-driven studies into large animals, given that the genetic profile, immune system, and the size and anatomy of the porcine spinal cord is recognized to better model the human^25^. Gӧttingen minipigs >15 kg (n = 6, 3 males and 3 females) were used in the present study. All animals underwent daily modified Tarlov-motor scoring to provide an ordinal scale of behavioral motor deficits for quantitative analysis (scored 0–9, Supplemental Table 1)^42^. All scoring was performed by investigators blinded to the side of injection and study design. Other clinical deficits may manifest due to a mass forming lesion in the thoracolumbar spinal cord. As such, physical examinations were performed by laboratory staff in consultation with veterinary staff daily, with both baseline and post-operative assessments until endpoint. All surgical procedures, animal care, endpoints, and the respective experiments on pigs were approved by the Emory University Institutional Animal Care and Use Committee (IACUC). All experiments were conducted with adherence to approved protocols set forth by the IACUC in coordination with the division of animal resources (DAR) and veterinary staff.

### Baseline and endpoint magnetic resonance imaging

The gold-standard for non-invasive tracking of tumor progression and recurrence of glioma in human patients is magnetic resonance imaging (MRI)^43^. To evaluate tumor growth, all animals underwent baseline and endpoint MRI scans. All scans were performed on a clinical Siemens 3T Trio MRI scanner with an integrated spine coil (Siemens Medical Solutions, Malvern, PA). Pre-contrast sequences included sagittal T1-weighted, T2-weighted, T1-fluid attenuated inversion recover (FLAIR) and T2-FLAIR sequences. Following intravenous gadolinium administration (0.1 mmol/kg, MultiHance-gadobenate dimeglumine, Bracco Diagnostics Inc.), post-contrast sequences were performed (sagittal and axial T1-FLAIR). Spatial saturation pulses were used for elimination of respiratory artifacts, and appropriate adjustments of field of view, flip angle, echo time, TR, and TI-interval were made. Radiologic features of interest included iso/hyper/hypo intensity, necrosis, contrast enhancement, rostro-caudal expansion, transverse cord involvement, tumor volume, spinal cord expansion, edema, and CSF obstruction. All scans were processed and representative images were acquired using ONIS-Dicom Viewer (DigitalCore, Co.Ltd, Tokyo, Japan).

### Tissue processing, H&E, and immunohistochemistry

At 3-week endpoint, all animals underwent euthanasia through intravascular administration of pentobarbital sodium and intracardiac perfusion with heparinized 0.9% saline solution. Spinal cords were harvested, post-fixed in 4% paraformaldehyde overnight, and cut into blocks at 5 mm intervals for paraffin embedding. Serial transverse spinal cord sections were cut on a microtome at 8um thickness for hematoxylin and eosin (H&E) and immunohistochemistry. H&E staining was performed with deparaffinization by serial xylene incubations and ethanol gradients in a standard fashion (Hematoxylin Gill No. 3, Sigma Aldrich, Cat: GHS332; Eosin Y, Sigma Aldrich, Cat: HT110132). Qualitatively, H&E sections were evaluated by a board-certified clinical neuropathologist (S.N. and P.C.) under wide-field microscopy blinded to the study design. Immunohistochemical stains were performed with primary antibodies for GFAP (Dako, Z0034), Olig2 (abcam, ab109186), Ki67 (abcam, ab15580) with appropriate secondaries for subsequent diaminobenzidine (DAB) and hematoxylin counterstaining (Thermo Scientific, Autostainer 480S). Immunohistochemical stains for glial markers (Olig2, GFAP) were qualitatively evaluated. Ki-67 index was quantified with manual counting of at least 1000 cells, scored as positive or negative. Representative images were acquired at 2x, 10x, 20x, and 40x magnifications (DS-Qi1 high sensitivity Cooled CCD camera, Nikon E400 microscope, NIS-Elements imaging software, Nikon Instruments, Inc) with calibrated scale bars prepared in ImageJ^44^. Whole slides were scanned in a raster pattern at 40x (Leica Aperio AT2 Slide Scanner).

### Data analysis and statistics

Descriptive statistics were used for all categorical variables, summarized as absolute and relative frequencies. Continuous and ordinal variables were summarized as appropriate using mean, standard deviation (SD), median and range. All clinical and behavioral analyses were performed by investigators blinded to the side of ONC or CTRL administration.

## Results

### Animals develop motor deficits ipsilateral to ONC injections by post-operative week 3

Post-operative behavioral analysis and physical examination was performed daily by blinded investigators until a 3-week endpoint. All animals developed clinically appreciable progressive motor deficits ipsilateral to the ONC injection, with 100% of animals (n = 6/6) developing hindlimb motor deficits by the 13^th^ post-operative day (Fig. 1C). 33% of animals (n = 2/6) progressed to develop bilateral hindlimb paralysis by the 3-week endpoint. At endpoint, Tarlov scores among animals ranged from 0–8, including either complete paralysis or impaired ambulation with the ability to walk greater than 1 minute respectively. Representative motor deficits are presented in Supplemental videos 1 and 2 taken at post-operative day 12 and 17 respectively in the same animal. Following tissue harvesting, appreciable mass-forming lesions were observable through the dura mater on gross pathology (Fig. 1C). No animals exhibited clinical signs of cachexia and the mean difference between baseline and endpoint body weight was 0.61 kg (SD: ± 0.94, Range: −0.5 to 2.1, Median: 0.55). A summary of hindlimb motor deficits, laterality, and clinical symptoms are outlined in Table 1. On gross examination, (Fig. 1D), a mass forming lesion was appreciated in 6/6 animals with appreciable expansion of the spinal cord. No tumoral or peritumoral cysts were appreciated.

**Table 1:** Overview of Clinical Findings in All Animals.

Animal	Baseline			Endpoint					
	Injection Site	Weight (kg)	mTS	Hindlimb Motor Deficits	Superficial Pain Response	Conscious Proprioception	Gastrointestinal Genitourinary	Weight (kg)	mTS
1	R: ONC	20.2	9	Right	R: Delayed	R: Delayed	WNL	20.4	8
	L: CTRL								
2	R: CTRL	19.7	9	Bilat	R: Absent	R: Absent	Urine Retention	21.8	0
	L: ONC				L: Absent	L: Absent			
3	R: ONC	15.3	9	Bilat	WNL	R: Absent	WNL	16.4	3
	L: CTRL					L: Absent			
4	R: CTRL	15.5	9	Left	WNL	L: Delayed	WNL	15.4	8
	L: ONC								
5	R: ONC	21.6	9	Right	R: Delayed	R: Absent	WNL	21.1	8
	L: CTRL								
6	R: CTRL	17.2	9	Left	WNL	WNL	WNL	18.1	8
	L: ONC								

Abbreviations: R – right, L – left, ONC – oncogenic vectors, CTRL – control vectors, Bilat – bilateral, GI/GU – Gastrointestinal or Genitourinary, WNL – within normal limits, mTS – modified Tarlov Score.

### Endpoint MRI demonstrates mass forming lesions consistent with high grade glioma

**Figure 2:**
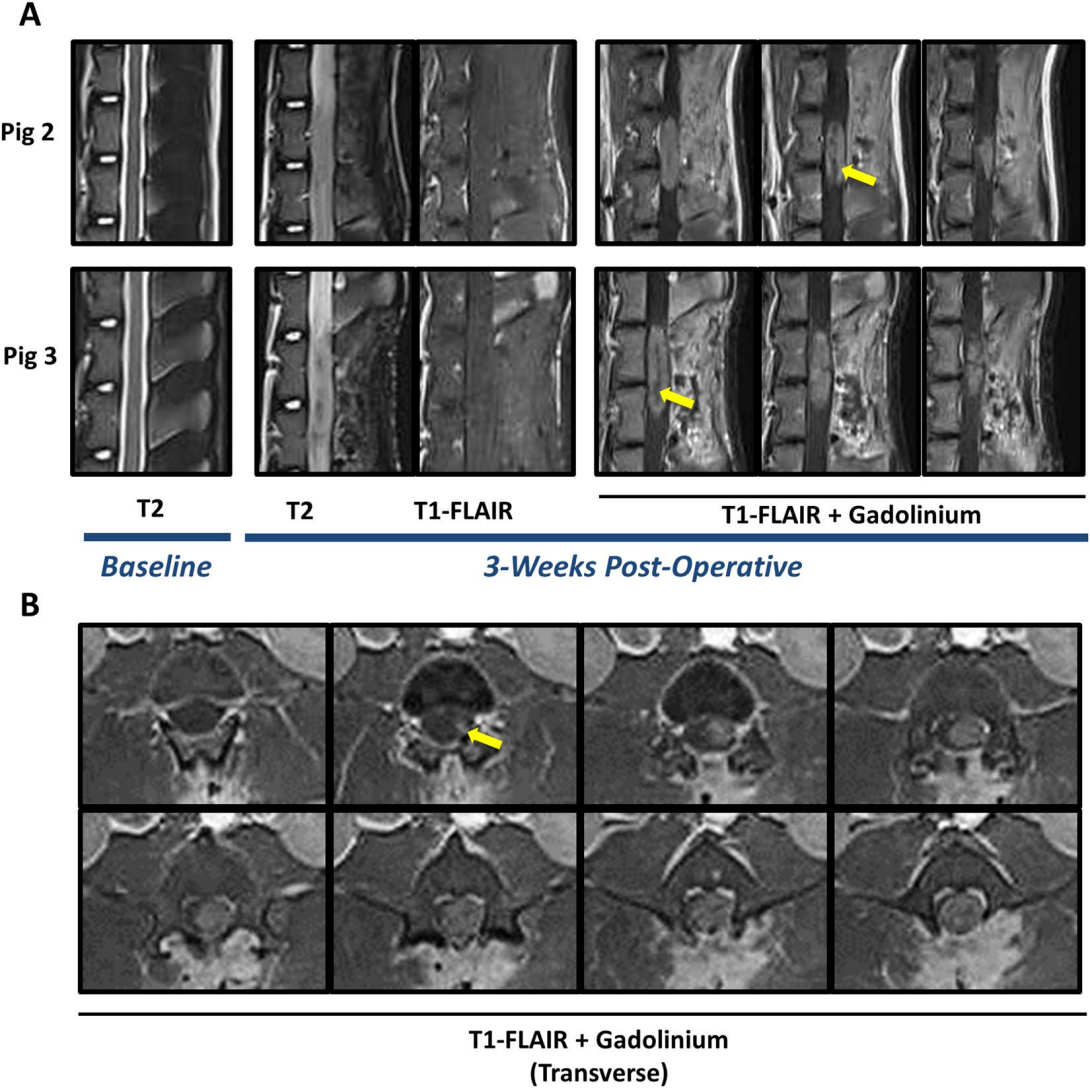
Endpoint MRI scans demonstrate mass forming lesions consistent with high-grade intramedullary spinal cord glioma. (**A**) Baseline T2 weighted scans demonstrate normal spinal cord anatomy. At 3-weeks post-operatively, T2 and T1-fluid attenuated inversion recovery (FLAIR) scans show mass forming lesions at the site of ONC injection in 6/6 animals (representative images of pig 2 and 3 shown). T2 weighted scans demonstrate cord expansion and obstruction of CSF, as compared to baseline scans. Sagittal T1-FLAIR post-contrast images show contrast enhancing mass with non-enhancing/heterogeneously enhancing core suggestive of necrotic foci (yellow-arrows). **(B)** Axial T1-FLAIR post-contrast images show complete cord involvement of contrast-enhancing mass forming lesion (yellow arrow demonstrates beginning of contrast enhancing lesion). Axial images spaced by 2.5 mm.

All 6 animals received baseline and endpoint MRI scans. Baseline imaging demonstrated normal spinal cord anatomy with unobstructed cerebrospinal fluid (CSF) pathways in the sub-arachnoid space that were visible on sagittal views. Representative images from two animals presented in Fig. 2A, T2, Baseline. 3-weeks post-operatively, T2 and T1-FLAIR scans demonstrated mass forming lesions at the location of ONC injections, but not CTRL injections, and appreciable cord expansion and obstruction of CSF **(**Fig. 2A, Post-Operative). No cysts, hemorrhage, or syrinx formations were observed. The mass forming lesions (6/6) localized to clinical motor deficits and the locations of ONC injections. Intravenous Gadolinium contrast was administered (0.1 mmol/kg) for post-contrast scans. Sagittal T1-FLAIR post-contrast scans showed contrast enhancing, invasive, mass forming lesions with up to two vertebral levels of rostro-caudal involvement **(**Fig. 2A, T1-FLAIR + Gadolinium, Post-Operative, Yellow Arrows). All animals developed radiologically consistent masses on MRI with T1 weighted isointensity, T2 iso/hyperintensity, and T1-post contrast enhancement. Transverse T1-FLAIR post-contrast images demonstrated degree of cord involvement in a representative animal**(**Fig. 2B, Yellow Arrow). Measurements on post-contrast axial and sagittal scans demonstrated a mean of 27.8 mm rostro-caudal invasion (SD ± 4.8; Range: 22.7 to 36.2; Median: 26.88) and a mean of 7.7 mm axial invasion (SD ± 1.2; Range: 6.1 to 9.0; Median: 8.1).

### Histopathologically confirmed high-grade glioma

**Figure 3:**
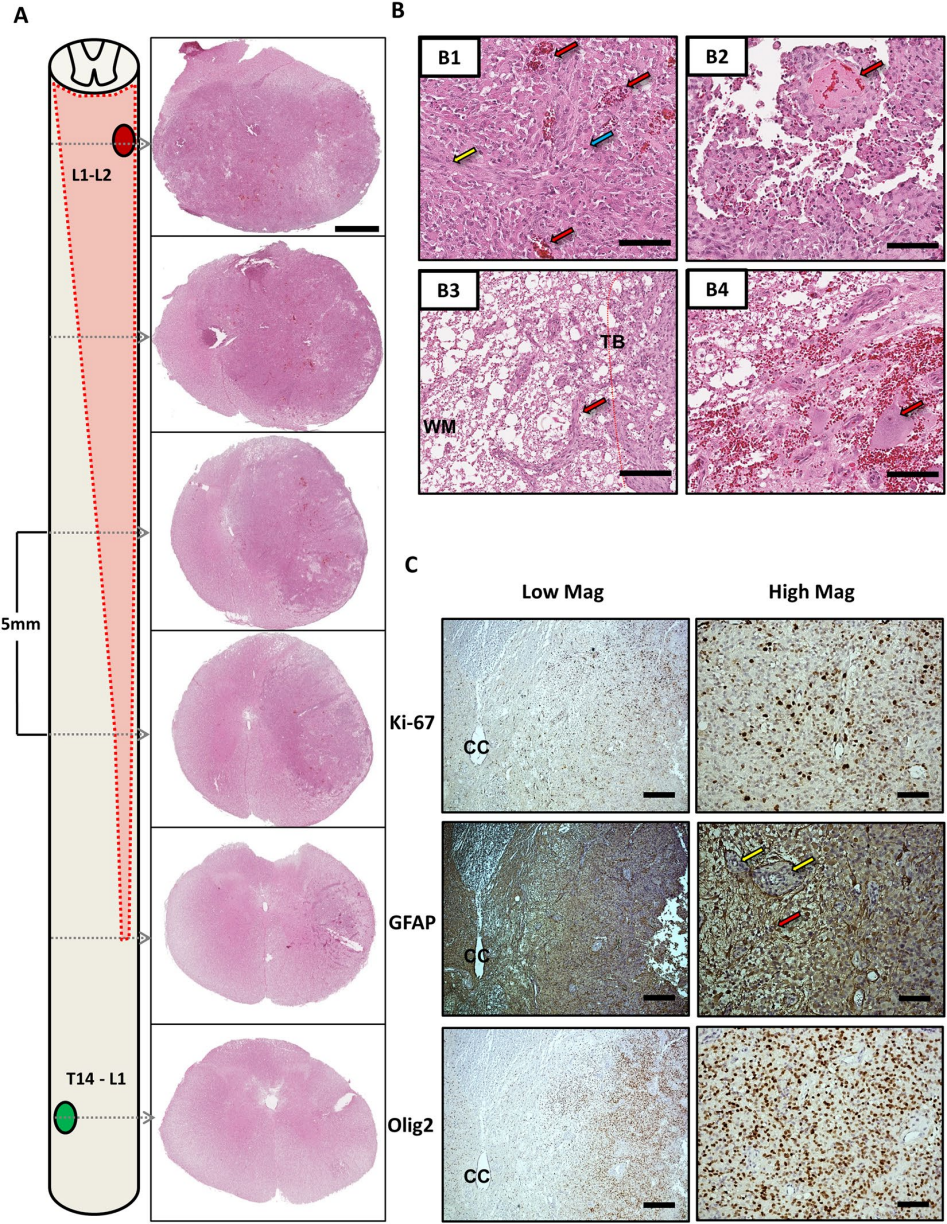
Histopathologic and Immunohistochemical Characterization. (**A**) Schematic of Tumor Growth and Low magnification H&E. Tumor growth occurred in 6/6 animals at the site of ONC injection (Red Oval) and not CTRL injections (Green Oval). Invasive growth was noted with frank rostro-caudal and transverse involvement (schematic, red dotted line and shaded region) and can be appreciated in serial sections with H&E staining demonstrating invasion along the lateral white matter (1x magnification, Scale Bar = 2 mm). The CTRL injection site did not demonstrate any morphological changes as depicted in the lowest low magnification panel (T14-L1). **(B)** High magnification tumor characteristics on H&E. Histopathologic features were investigated at high magnification, noting high cellularity with gemistocytic (Blue Arrow) and fibrillary (Yellow Arrow) astrocytic morphology (Panel B1), microvascular proliferation (B1, Red Arrows), and regions of necrosis and thrombotic blood vessels (B2, Red Arrow). Invasion along the tumor border (TB) into surrounding white matter (WM) (B3, Red arrow). In addition, background parenchyma was visible in regions of the tumor, here depicting motor neurons of the ventral horn (B4, Red Arrow). (40x magnification, Scale Bar = 100um). **(C)** Immunohistochemical Staining for Glial Markers and Proliferative Index. Standard fixed, paraffin embedded staining protocols with citrate mediated antigen retrieval were applied to 8um thick serial sections (n = 6–8 sections/animal/stain). GFAP staining demonstrated highly positive staining in the tumor mass (Red Arrow) with intervening negative staining of vasculature (Yellow Arrows). Olig2 demonstrated highly positive regions within the tumor mass. Ki-67 showed a high proliferative staining with a mean proliferative index of 37.1% (SD: ± 14.2). (Left: 4x magnification, Scale Bar = 500 um, Right: 20x magnification, Scale Bar = 100 um. CC: Central Canal).

On H&E staining at low magnification, the tumors diffusely infiltrate the spinal cord, spreading rostral and caudal with respect to the injection site, and invading both grey and white matter. There is noticeable mass effect on the central canal and contralateral structures (Fig. 3A) in all animals. In addition, there is marked invasion along the white matter tracks with cellular regions showing hyperchromasia visible in sections distal to the site of injection (Fig. 3A). Histopathologic features identified at high magnification include: high cellularity with epithelioid, and fibrillary astrocytic morphology, microvascular proliferation, necrosis, thrombosed blood vessels, and invasion along the tumor border into surrounding normal parenchyma **(**Fig. 3B**)**. Subsequent evaluation by board-certified neuropathologists (S.N. and P.C.) resulted in confirmation of the mass forming lesions as high-grade gliomas with astrocytic morphology, with consistent features in all animals. Ki-67 staining was highly positive across animals, (Fig. 3C) with a mean proliferative index of 37.1% (SD: ± 14.2). In addition, immunohistochemical staining was highly positivity for glial markers GFAP and Olig2 (Fig. 3C).

## Discussion

The data of the present study illustrates the first lentiviral vector induced large animal model of high-grade glioma with histopathologic, radiologic, and behavioral characterization. We found that high-grade gliomas formed in a highly penetrant fashion following the injection of ONC lentiviral vectors and exhibited histopathologically confirmed features of high-grade gliomas. Importantly, this has not been performed in large animal models prior to this study and we assert that this paradigm using intraparenchymal gene transfer may be adapted for use in spinal cord, brainstem, cerebellum, and brain modeling of glioma.

Current preclinical studies for therapeutic approaches including, but not limited to, oncolytic viral therapy, immunotherapy, convection-enhanced delivery, fluorescein dyes and laser interstitial thermal therapy in high-grade glioma rely on large animal models with no ability to evaluate therapeutic efficacy in disease models^7^. By using orthotopic intraparenchymal gene transfer in higher-order species, given the advantages of an immunocompetent porcine model, investigators can reasonably adjust transgenes and/or promoters to dissect the disease process and develop translationally relevant systems in a variety of glioma grades and subtypes.

Utilizing gene transfer in pigs as a system for modeling tumors in the CNS offers several advantages. For one, this approach has been previously employed in porcine CNS gene transfer for other neurologic disease modeling and can be widely scaled for implementation and adaptation in wildtype pigs for use in preclinical therapeutic development programs^45,46^. Importantly, this would circumvent the need for generation and maintenance of numerous transgenic breeds, providing a pathway for rapid induction in widely available pigs. In addition, when using lentiviral gene transfer – third or second generation replication deficient lentiviruses permit large insert size, minimal immunogenicity, and a broad tissue tropism with a VSV-G pseudotype^38^. Furthermore, lentiviral genomic integration into the transduced cell permits the continued expression in daughter cells of the proliferative environment of an induced glioma^16^. As such, examination of the most common patient-population and respective patterns of mutations may allow for targeted modeling with ease of inclusion or exclusion of specific genetic lesions (e.g. loss of ATRX, IDH1 mutations, EGFRvIII, PTEN, p53, H3K27M) with multiple combined constructs. Consequently, this paradigm could provide a foundation for utilizing specific transgenes, promoters, and titer for a host of potential glioma grades, subtypes, and other CNS tumors in porcine models. This strategy can be reasonably implemented using non-viral systems (sleeping beauty transposon system) or other vector driven gene editing approaches (CRISPR) to develop an armamentarium of immunocompetent translational animal model systems for neoplasia of the central nervous system. By drawing from clinical insights, applying foundational research, and validating response to the clinical standard of care, we assert that such a modeling approach would provide a highly impactful space for both bench science and translational therapeutic initiatives.

### Limitations and future directions

There are several areas that warrant investigation in future studies. One area of interest is whether these tumors progress as the result of clonal expansion of virally infected cells, or through the recruitment and transformation of resident glial progenitors^15,16,22^. In addition, it is necessary to evaluate tumor biopsies for accumulated mutations that occur in the environment of p53 knockdown, PDGF-B expression and HRAS-G12V expression, and an overall picture of proliferative stress^47^. As such, the importance of sequencing tumor biopsies for evaluation of copy-number variation, single-nucleotide polymorphisms and mutational insertions or deletions cannot be overstated. The rise of molecular subtyping have provided putative categories for glioblastoma and high-grade glioma^48,49^, which have marked inter-tumoral and intra-tumoral heterogeneity in patients^50,51^. To validate such models with respect to these subtypes and evaluate the degree of inter- and intra-tumoral heterogeneity, the use of bulk and single-cell RNA sequencing for transcriptomic investigation is warranted. These data can be readily compared to our existing understanding of the disease and open-source patient data sets such as The Cancer Genome Atlas (TCGA) as has been conducted in prior studies. Furthermore, it is critical from a translational standpoint to study the use of surgical resection, associated strategies and challenges, and the characterization of tumor recurrence in the present model. Lastly, the characterization of host immunologic responses in the swine in response to these lentiviral injections will need to be understood for testing and understanding immunotherapeutic interventions. Limitations and future directions notwithstanding, the present study describes the first histologically, radiologically, and behaviorally characterized immunocompetent porcine spinal cord glioma model.

## Conclusion

Lentiviral gene transfer of driver mutations represents a feasible pathway to modeling glioma in higher order species. Here we report the first lentiviral induced large animal model of high-grade spinal cord glioma. This can be potentially used in a variety of preclinical therapeutic development programs.

## Supplementary information

Supplementary Information.

Supplementary Information 2.

Supplementary Information 3.

Supplementary Information 4.

Supplementary Information 5.

Supplementary Information 6.
