# Supplementary figures and images for: Lentiviral Vector Induced Modeling of High-Grade Spinal Cord Glioma in Minipigs
Source: Sci Rep. 2020 Mar 24;10:5291. doi: 10.1038/s41598-020-62167-9 (PMC7093438; doi:10.1038/s41598-020-62167-9)

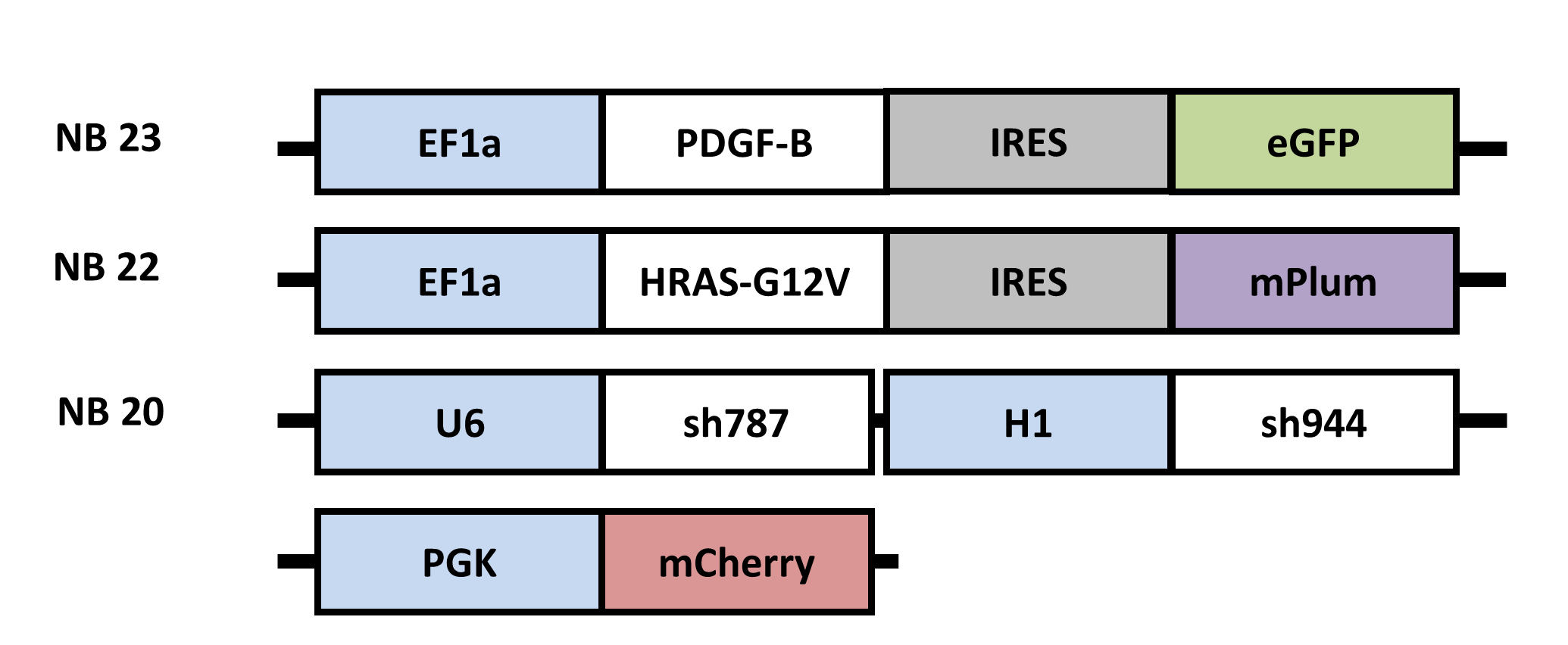

Supplement: Supplementary file 1 — Supplementary Information. [file 41598_2020_62167_MOESM1_ESM.tif]
