# Supplementary material for: Lentiviral Vector Induced Modeling of High-Grade Spinal Cord Glioma in Minipigs
Source: Sci Rep. 2020 Mar 24;10:5291. doi: 10.1038/s41598-020-62167-9 (PMC7093438; doi:10.1038/s41598-020-62167-9)
Supplement: Supplementary file 2 — Supplementary Information 2. [file 41598_2020_62167_MOESM2_ESM.docx]

| **Supplemental Table 1: Criterion for modified Tarlov Scoring (mTS) used in Gait Assessments** | |
| --- | --- |
| **mTS** | **Description of Criteria** |
| **9** | normal HL motor function |
| **8** | ability to walk > 1 minute |
| **7** | ability to walk < 1 minute |
| **6** | ability to get up and stand unassisted more than 1 minute |
| **5** | ability to get up with assistance and stand unassisted more than 1 minute |
| **4** | ability to get up with assistance and stand unassisted less than 1 minute |
| **3** | ability to get up and stand with assistance less than 1 minute |
| **2** | good movements at joints but inability to stand |
| **1** | perceptible movements at joints |
| **0** | no voluntary movements |
