# Supplementary material for: Lentiviral Vector Induced Modeling of High-Grade Spinal Cord Glioma in Minipigs
Source: Sci Rep. 2020 Mar 24;10:5291. doi: 10.1038/s41598-020-62167-9 (PMC7093438; doi:10.1038/s41598-020-62167-9)
Supplement: Supplementary file 6 — Supplementary Information 6. [file 41598_2020_62167_MOESM6_ESM.docx]

**Supplemental Figure 1: Schematic of vector design.** Lentiviral vectors targeting the RTK/RAS/Pi3K and p53 pathways were designed separately to avoid the risk of decreased transduction efficiency from larger insert sizes, safety considerations. All lentiviral vectors are third-generation, replication deficient systems. Two of the lentiviral vectors used a pCDH transfer plasmid backbone with a ubiquitous Ef1α promoter and fluorescent reporters following an internal ribosomal entry site (IRES) sequence. These included Vector 1: PDGF-B-IRES-eGFP and Vector 2: HRASG12V-IRES-mPlum. Vector 3 was designed using a pLKO1 backbone and expressed two sequences of shRNA targeting porcine p53 including sequences 787 and 944 under the H1 and U6 promoters, respectively. These sequences have 100% homology to two regions of porcine p53 mRNA and have been used for efficient knockdown in porcine cell-culture models [12]. For a fluorescent reporter in this vector, the PGK promoter was included with expression of mCherry. Controls for vectors 1-3 only included fluorescent probes and shRNA scramble, herein referred to as CTRL. The oncogenic cocktail will be referred to as ONC.
